# Presumed adverse events in health care are a frequent indication for medico-legal autopsy in Finland

**DOI:** 10.1007/s12024-019-00193-4

**Published:** 2019-11-18

**Authors:** Lasse Pakanen, Noora Keinänen, Paula Kuvaja

**Affiliations:** 1 Forensic Medicine Unit, National Institute for Health and Welfare (THL), P.O. Box 310, FI-90101 Oulu, Finland; 2 Department of Forensic Medicine, Research Unit of Internal Medicine, Medical Research Center Oulu, University of Oulu, P.O. Box 5000, FI-90014 Oulu, Finland; 3 Department of Pathology, Oulu University Hospital, P.O. Box 50, FI-90029 – OYS Oulu, Finland

**Keywords:** Medico-legal autopsy, Adverse event, Clinical risk management, Malpractice, Medical error

## Abstract

The medico-legal autopsy is an essential tool in investigating deaths caused by an adverse event in health care, for both clinical risk management and for professional liability issues. However, there are no statistics available regarding the frequency of autopsies performed due to suspected adverse events. This study aimed to determine the number of medico-legal autopsies done because of presumed adverse events, whether these events were unintentional, medical errors or cases in which malpractice was suspected. Furthermore, differences in treatment types, causes and manner of death were analyzed. The data was obtained from all medico-legal autopsies performed in Northern Finland and Lapland during 2014–2015 (*n* = 2027). Adverse events were suspected in 181 (8.9%) cases. The suspicions of an adverse event occurring were most often related to medication, gastrointestinal surgery and orthopedic surgery. The manner of death was classified as *medical (or surgical) treatment or investigative procedure* in 22 (12.2%) cases. The causes of death were completely unrelated to the suspected adverse event in 41 (22.7%) cases. In conclusion, the frequency of presumed adverse events was quite high in this data set, but in the majority of the cases, the suspicion of an adverse event causing death was disproved by an autopsy. Nonetheless, proper investigation of these cases is essential to ensure legal protection of the deceased, next of kin and health care personnel, as well as to support clinical risk management.

## Introduction

Mortality related to health care is a major concern considering patient safety and the quality of treatment. The importance of this topic has grown constantly as a result of public awareness and expectations towards medical science [1]. However, there are no exact statistics available about treatment-related mortality or the number of deaths in which an adverse event or error are suspected. Some estimations of the number of deaths caused by medical errors have been issued for different countries [2–5], and it has been proposed that medical errors comprise the third leading cause of death in the United States [6]. The definition of such events is complex [7, 8], which makes giving precise estimations a difficult task.

Deaths caused by health care can be divided into (unpreventable) adverse events, preventable adverse events and negligent adverse events (malpractice) [9]. Adverse events are caused by unintended reactions to treatment, for example, a medication-related reaction or an unavoidable post-surgical infection. A preventable adverse event can be related to human or system factors. Malpractice is caused by neglect, either intentional or unintentional, by personnel involved in the duty of care.

**Fig. 1:**
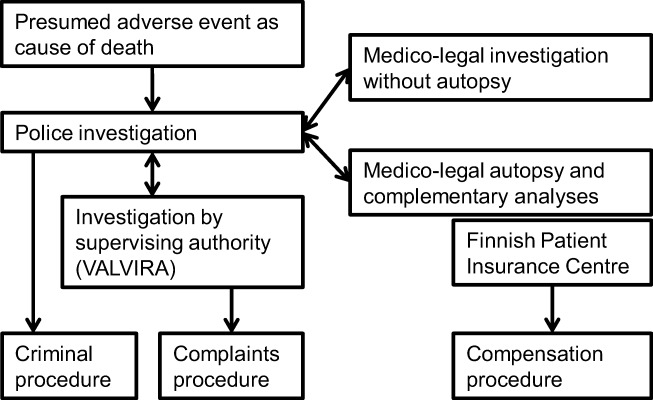
The investigation process when an adverse event is presumed as a cause of death in Finland. The National Supervisory Authority for Welfare and Health (VALVIRA) handles the complaints procedure which can be initiated by the police or directly by the deceased’s next of kin. Based on the statement of the supervising authority, the police may also initiate a criminal investigation. Issues concerning financial compensations are handled by another independent authority, the Finnish Patient Insurance Centre. These three procedures do not exclude one another and can be claimed irrespective of each other

Medico-legal cause-of-death investigation is an essential part of determining treatment-related fatalities [10–12]. Considering cases of medical error and malpractice, there is large variation in how suspected or confirmed cases are reported [2, 13]. In order to align standards and evaluation methods, the European Academy of Legal Medicine has issued guidelines on the ascertainment of medical liability cases [11]. From the standpoint of clinical risk management, the focus on adverse events is usually not as conspicuous in the medico-legal setting. According to Finnish law, a police investigation is mandatory if a person is suspected or presumed to have died from an adverse event of any kind (the Act relating to cause-of-death investigation 459/1973), and usually a medico-legal autopsy ensues. This is to ensure the proper investigation of deaths presumed to have been caused by health care. The suspicion can be raised by the deceased’s relatives, the health care personnel involved, the police or other authority. After consultations between the police, forensic pathologist and clinicians, the mode of the cause-of-death investigation is decided. Followed by the police investigation and medico-legal autopsy, different procedures can be claimed depending of the nature of the adverse event. These procedures are handled by various authorities, and they do not exclude one another (Fig. 1).

The causes of death are reported in many countries according to the International Statistical Classification of Diseases and Related Health Problems 10th Revision (ICD-10) [14]. In addition, the Finnish cause-of-death statistics include a unique manner of death, namely *medical (or surgical) treatment or investigative procedure,* which is not included in the World Health Organization classification. This is a neutral term used for cause-of-death statistics, and it does not indicate whether the adverse event has been due to an unintended reaction, medical error or neglect.

Experience shows that treatment procedures, most often surgical ones, undertaken prior to death are quite often presumed to have contributed to an adverse patient outcome, but are only found to be significant in a small proportion of these cases. However, it has not been assessed how many resources are used in forensic medicine to investigate the possible adverse events of health care. In this study, we aimed to determine the frequency of medico-legal autopsies performed because of presumed adverse events of any kind in health care. The proportion of deaths in which the manner of death was classified as *medical (or surgical) treatment or investigative procedure* was also assessed. Furthermore, we wanted to analyze what treatment types were most often associated with the suspected adverse events, and whether there were any statistical differences between different groups. The study is part of a larger study protocol concerning deaths due to health care related adverse events in Finland.

## Materials and methods

### Data collection

Data was gathered from autopsy reports, death certificates and police reports related to medico-legal autopsies carried out in the National Institute for Health and Welfare, Oulu, Finland, during 2014–2015. The autopsy data consisted of deaths that had occurred in Northern Finland and Lapland (*n* = 2027). This constitutes 11% of the medico-legal autopsies performed in Finland as a whole during the study period. Based on police reports and related documents, all cases where the indication for medico-legal autopsy had been a suspected adverse event in health care were selected. Permission to gather data from the documents of medico-legal death investigation was obtained from the National Institute for Health and Welfare (Dnro: THL/1078/6.02.00/2017).

Gender, age, causes of death and manner of death were analyzed. The cases were grouped according to the type of medical treatment or surgical procedure administered prior to death as follows: 1) medication-related adverse event; 2) gastrointestinal surgery, including endoscopic retrograde cholangiopancreatography, percutaneous endoscopic gastrostomy, and liver biopsies, excluding routine gastro- or colonoscopy; 3) arterial surgery and large vein catheters, including procedures on the carotid arteries, excluding intracranial procedures as well as heart and thoracic surgery; 4) orthopedic surgery; 5) urological surgery; 6) cardiothoracic surgery, including lung biopsies and surgical procedures on airways; 7) standard, smaller procedures, including routine gastro- or colonoscopy and thoracocentesis and/or pleural fluid drainage; 8) gynecological surgery; 9) neurosurgery and intracranial artery operations; 10) cardiological operation such as angiography, transcatheter procedures and pacemaker-related events, excluding surgical operation included in group 6; 11) other medical or surgical treatment, including radiation therapy, hemodialysis and rejection reactions and unspecified suspicions.

### Statistical analyses

Statistical analyses were performed with IBM SPSS Statistics version 25 (Armonk, NY, USA). Nonparametric tests were used to determine statistical differences, and median and range are presented.

## Results

### Demographic data

The median age (range) was 77 (0–98) years. The highest median age was in the orthopedic surgery group (84, 55–96 years) and the lowest in the group of other and unspecified procedures (69, 0–90 years). The difference in median ages was statistically significant between the groups (*p* = 0.019, Kruskal-Wallis test), but not according to the manner of death (*p* = 0.063, Kruskal-Wallis test). The gender distribution was 97 (53.6%) men and 84 (46.4%) women. There were no significant differences according to sex.

### Suspected adverse events as indication for medico-legal autopsy

The indication for medico-legal autopsy was a suspected health care related adverse event in 181 cases (8.9% of all medico-legal autopsies in Northern Finland and Lapland in 2014–2015). The indication was clearly specified in the police report in 131 (75.7%) cases. In the remaining cases, the indication was discernible from the related documents (e.g. clinician’s autopsy request).

**Fig. 2:**
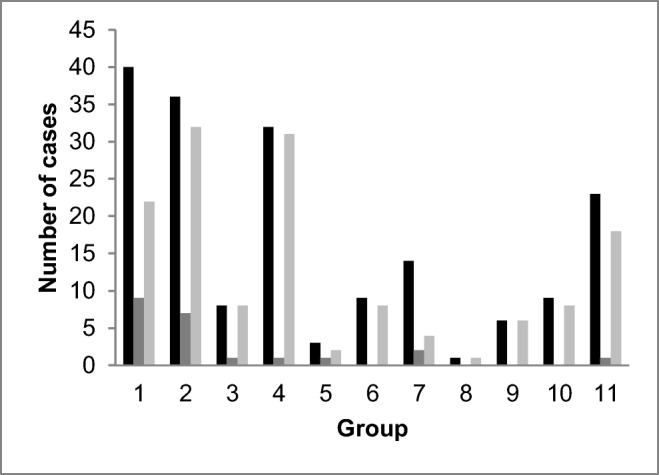
The number of presumed adverse events (black bars) and cases with the manner of death classified as *medical (or surgical) treatment or investigative procedure* (dark grey bars) in different treatment groups. The light grey bars represent cases in which the cause(s) of death were somehow related to the presumed adverse event. The groups are: 1, medication-related; 2, gastrointestinal surgery; 3, arterial surgery; 4, orthopedic surgery; 5, urological surgery; 6, cardiothoracic surgery; 7, standard small procedures; 8, gynecological surgery; 9, neurosurgery; 10, cardiological operation; 11, other and unspecified

The suspected adverse events were most often related to medication (*n* = 40, 22.1%), gastrointestinal surgery (*n* = 36, 19.9%) and orthopedic surgery (*n* = 32, 17.7%) (Fig. 2). The types of medical or surgical treatment suspected to have caused the death are summarized in Table 1.

**Table 1:** Presumed adverse events in different treatment categories and the classification of deaths

Treatment categories in presumed adverse events	Manner of death									
	Disease		Medical treatment or investigative procedure		Accident		Undetermined		Total	
	n	%	n	%	n	%	n	%	n	%
1) Medication-related	28	70.0	9	22.5	0	0.0	3	7.5	40	22.1
2) Gastrointestinal surgery	29	80.6	7	19.4	0	0.0	0	0.0	36	19.9
3) Arterial surgery	7	87.5	1	12.5	0	0.0	0	0.0	8	4.4
4) Orthopedic surgery	20	62.5	1	3.1	11	34.4	0	0.0	32	17.7
5) Urological surgery	2	66.7	1	33.3	0	0.0	0	0.0	3	1.7
6) Cardiothoracic surgery	9	100.0	0	0.0	0	0.0	0	0.0	9	5.0
7) Standard small procedures	12	85.7	2	14.3	0	0.0	0	0.0	14	7.7
8) Gynecological surgery	1	100.0	0	0.0	0	0.0	0	0.0	1	0.6
9) Neurosurgery	4	66.7	0	0.0	2	33.3	0	0.0	6	3.3
10) Cardiological operations	9	100.0	0	0.0	0	0.0	0	0.0	9	5.0
11) Other and unspecified	17	73.9	1	4.3	4	17.4	1	2.2	23	12.7

### Causes and manner of death

The manner of death was classified as *medical (or surgical) treatment or investigative procedure* in 10 (10.8% of yearly suspected cases) and 12 (13.6%) cases in 2014 and 2015, respectively (Fig. 2). Additionally, there were a total of 138 (76.2% of all suspected cases) disease deaths, 17 (9.4%) accidental death, and 4 (2.2%) deaths in which the manner of death was undetermined.

The majority of deaths caused by adverse events were related to medication (*n* = 9, 5.0%) and gastrointestinal surgery (*n* = 7, 3.9%). The differences between groups were statistically significant (*p* = 0.001, chi-square test), but the number of cases was too small in some groups to draw conclusions. The causes of death were mostly hemorrhages and perforations (*n* = 10, 5.5%) as well as medication-related adverse effects and intoxications (*n* = 9, 5.0%). Additionally, there was one postsurgical wound infection, one fat embolization after the application of a joint prosthesis and one complication related to pregnancy and delivery. The most frequent cause of death among all cases was cardiovascular disease (*n* = 102, 56.4%).

In the majority of cases, the causes of death were linked to the initially suspected treatment or procedure (*n* = 130, 71.2%); for example, coronary artery disease as cause of death after angiography (Fig. 2). In 41 (22.7%) cases, there was no relation between the causes of death and the initial suspicion, and in 10 (5.5%) cases this could not be assessed because the suspicion was not specified. Groups 1 (medication-related) and 7 (standard small procedures) had the highest proportion of cases in which the suspected treatment and cause of death were unrelated, 71.4% and 45.0%, respectively. The differences between groups were significant (*p* < 0.001, chi-square test), but some groups were too small to draw conclusions.

## Discussion

The percentage of medico-legal autopsies in which there was a suspected adverse event was surprisingly high (8.9%). Extrapolating this result to include the whole country, this would mean that annually more than 800 autopsied death cases were suspected to have been caused by an adverse event. This percentage was twice as high as that reported in Germany where 4.4% of all medico-legal autopsies were done because of medical malpractice claims [5, 10]. One reason for the higher number in Finland is that the threshold for performing a medico-legal autopsy has traditionally been kept low when a health care related adverse event is suspected. These cases also include adverse events that are not considered malpractice, but are caused, for example, by systemic errors. The investigation of these deaths are considered important from the point of view of clinical risk management. Also, all these cases are thoroughly investigated to ensure the legal protection of all parties considering possible malpractice claims. Therefore, the request for medico-legal autopsy can be brought forward by the treating physician, the police or the deceased’s next of kin.

Deaths in which the manner of death was classified as *medical treatment or investigative procedure* were quite uncommon, as was expected. Based on these figures, there would be about 100 deaths caused by adverse events in Finland every year, although this estimation has to be confirmed in subsequent studies. However, it is obvious that the number of deaths caused by adverse events falls far below the previous estimations of 700–1700 yearly deaths from medical errors [3, 4].

Besides medication-related adverse effects, the adverse event suspicions were mainly related to different surgical disciplines. This was expected and in line with previous studies [5, 15, 16]. It is notable that the confirmed treatment-related fatalities were almost exclusively related to medication and gastrointestinal surgery. Orthopedic procedures were often presumed as cause of death, but there was only one confirmed case, even though, interestingly, mishaps in hip and knee arthroplasty procedures are among the adverse events most frequently compensated by the Finnish Patient Insurance Centre [17]. There were no confirmed cases related to cardiothoracic surgery, cardiological operations, neurosurgery and gynecological surgery in this study population. Furthermore, in every fifth suspected case, the determined causes of death were totally unrelated to the initially claimed suspicion. The suspicions were disproven in 71.4% of the standard small procedures, including endoscopic examinations and puncturing procedures.

The frequency of autopsies in Finland has traditionally been high [18], but in recent years the need for such a high number of medico-legal autopsies has been debated [19], and some decline in the autopsy rate has already been observed. Although it seems that many of the presumed adverse event cases were uncalled for, we have to be careful not to exclude these cases from undergoing medico-legal autopsies. Besides diagnosing the causes of death, the autopsy also serves as part of the quality control system in health care [11]. Furthermore, presumptions and suspicions of medical errors and downright malpractice are likely to increase in the future with the rising awareness and expectations of the public [1].

Our data included all presumed adverse events, whether caused by unintentional reactions, errors or neglect. In the medico-legal literature and public discussions, most interest has, understandably, been in the malpractice cases [2, 10]. The investigation of malpractice claims should be done carefully following a methodological approach, and in some countries, the role of the forensic pathologist may extend further than the actual cause-of-death investigation [11]. In Finland, the medico-legal autopsy serves as a tool for both clinical risk management and professional liability issues. However, when necessary, different authorities assess whether the treatment given has been appropriate or errors have been made (Fig. 1). To get a comprehensive impression of health care related mortality in Finland, the confirmed malpractice cases need to be evaluated in more detail.

## Conclusions

In the medico-legal autopsy material included in our study adverse events were quite often presumed to have caused death. The number of confirmed cases was, however, low. Nonetheless, it is essential to investigate these deaths thoroughly: on the one hand, to avoid unjustified accusations and on the other hand, to enhance patient safety protocols. According to the results of our study previous estimations about Finnish health care related mortality seem to be grossly overestimated, but this has to be confirmed in a more comprehensive setting.

## Key points

1. Statistics about the frequency of autopsies performed because of deaths caused by presumed adverse events in health care are not available.
2. The number of medico-legal autopsies done because of presumed adverse events, including adverse events from unintentional reactions, medical errors or malpractice was determined.
3. Adverse events were suspected to have contributed to death quite frequently, but the suspicion was confirmed in only 12.2 % of studied cases.
4. The suspicions were most often related to medication, gastrointestinal surgery and orthopedic surgery, and the confirmed cases were mostly related to medication and gastrointestinal surgery.
5. The causes of death were completely unrelated to the suspected event in more than every fifth case.
